# Storage media for computers in radiology

**DOI:** 10.4103/0971-3026.43838

**Published:** 2008-11

**Authors:** Ravi Varma Dandu

**Affiliations:** Department of Radiology, Krishna Institute of Medical Sciences, Secunderabad, India

**Keywords:** Storage media, computers

## Abstract

The introduction and wide acceptance of digital technology in medical imaging has resulted in an exponential increase in the amount of data produced by the radiology department. There is an insatiable need for storage space to archive this ever-growing volume of image data. Healthcare facilities should plan the type and size of the storage media that they needed, based not just on the volume of data but also on considerations such as the speed and ease of access, redundancy, security, costs, as well as the longevity of the archival technology. This article reviews the various digital storage media and compares their merits and demerits.

Since the pioneering days of radiology, film-based radiography has been the principal mode of capturing, viewing, and storing radiological information. Though the radiographic film continues to be the most commonly used medium for storing imaging information, it has the disadvantages of being expensive; difficult to duplicate; and cumbersome to transport, store, and retrieve. There is also the possibility of data loss due to physical deterioration of the film over time.

Over the last 25 years, imaging techniques such as CT scan and MRI, which are based on digital technology, have been introduced and have found widespread application in medical imaging. These studies allow physicians to examine the body in minute detail and enable rapid and accurate diagnosis of disease states. In contrast to conventional radiography, these techniques usually produce hundreds or even thousands of images per study. As the benefits of digital images have become apparent, imaging modalities that have traditionally been analog based have also begun evolving into digital systems. Computed radiography, digital fluoroscopy, digital mammography, and USG are examples of modalities that have benefited from the introduction of digitization into the imaging chain.

These changes in radiology workflow in the recent years have resulted in a virtual explosion in the amount of image data produced by radiology departments [Table 1]. Though the entire data is required by the radiologist or physician who interprets the study, it would be impossible to archive such large volumes of image data by printing on film.

**Table 1:** File sizes of images from different imaging modalities

Modality	Image matrix (in pixels)	Dynamic range (bits per pixel)	File size (per image)
MRI	256 × 256	16	131 KB
CT Scan	512 × 512	16	524 KB
Ultrasound	512 × 512	8	262 KB
Color Doppler	768 × 576	8	442 KB
Digital radiography	Up to 3000 × 3000	Up to 16	Up to 18 MB
Digital mammography	Up to 3328 × 4096	14	27 MB
Computed radiography	3520 × 4280	12	30 MB

Table modified from reference 8

In addition to images from the radiology department, large quantities of data from the cardiology department or other imaging data such as clinical photographs and microscopic and endoscopic photographs also find their way into the image database. Thus, as hospitals move towards a filmless, paperless environment, there will be a never-ending demand for digital storage space.[1]

The introduction of picture archiving and communications systems (PACS) for archival, migration, and display of digital images has resulted in increased productivity by expediting image-based workflow. The data storage system is the heart of the PACS system and, most often, its costliest component.[2] A reliable data storage system with a large capacity, which provides immediate access to the entire imaging archive with minimal operator intervention, forms the foundation of any PACS installation.[3] We shall review the various types of media that are available for storage of image data in the PACS environment.

## Storage media

Storage can be classified as online, nearline, and offline. Online storage refers to data storage on magnetic discs and redundant array of inexpensive discs (RAID) systems. It provides access to the data in a few milliseconds. As this type of storage is expensive, images that do not require immediate access are stored in nearline storage. Devices such as magnetic tapes and optical jukeboxes are used for this form of storage. Offline storage media include magnetic tapes and optical discs that are stored elsewhere. This type of storage is typically used for long-term storage and for storing back-up data.

### 1. Magnetic disc

The magnetic disc (hard disc drive) offers the fastest way to store and access large amounts of data. The data is physically stored by inducing magnetic moments on a ferromagnetic disc as it is spinning. The read–write head detects and modifies the data on the disc platter. The speed of rotation of the disc and the density of information on the disc head determine the physical performance of the disc.

### 2. Redundant array of inexpensive discs

Redundant array of inexpensive discs (RAID) is a series of hard discs plugged together using shared logic to act like a single large disc. It aims to achieve a large data storage capacity with better input/output functionality, while minimizing cost and maximizing reliability by using redundancy of information. Data is subdivided into multiple consecutive segments that are distributed over several physical discs by a fast controller card. The data can be mapped using various patterns so as to achieve various levels of speed, capacity, and protection against data loss. RAID configurations that are commonly employed include RAID level 0, RAID level 1, and RAID level 5.[4]

- RAID level 0 involves the spread of information onto all hard discs, without any redundancy. This architecture improves speed and makes use of the maximum capacity of the discs; however, a single disc failure may result in the loss of all data on the RAID.
- RAID level 1 creates an exact copy of the data on multiple discs. As all data is duplicated, the array continues to function as long as at least one disc is functioning. Though this architecture has the best reliability, the storage capacity of the array is limited to the size of one single disc within the array.
- RAID level 5 is an efficient balance of performance and reliability. It involves storage of ‘parity’ information on each disc that permits reconstruction of lost data. The information needed for reconstruction occupies less space than simple mirror imaging of the data. As the parity data occupies the size of one disc in the array, the total storage capacity of the array is reduced by one disc.

Current PACS technology largely relies on RAID to provide large storage spaces and fast access times.

### 3. Optical discs and magneto-optical discs

Optical discs have been the medium of choice for archiving data for many years.[5] A number of small hospitals and imaging facilities still use this medium for archival. Most optical media such as CDs and DVDs are cheap and can be read in most personal computers without the need for additional dedicated disc drives. The major disadvantages of optical media are the relatively low capacity per disc, low data transfer rates, and poor reliability. These discs are unprotected and are prone to physical damage and data loss. Magneto-optical discs offer higher storage capacities but are slower in writing and accessing data.

### 4. Magnetic tape

Magnetic tape provides the cheapest option for storing large amounts of data. It has higher readout speeds than optical and magneto-optical media. However, since data is read from the tape using sequential access (while data readout from magnetic discs is by random access), identification of individual scans and patients is typically much very slow. Of late, magnetic tapes are being used only as media for providing offline backup of data.

The characteristics of these different storage media are compared in Table 2.

**Table 2:** Comparison of digital storage media

	Magnetic discs	Optical media	Magnetic tape
Access	Random access	Sequential access	Sequential access
Storage capacity	Up to 300 GB	Up to 30 GB	Up to 500 GB
Write speed	Fast	Slow	Slow
Read speed	Fast	Fast	Slow
Study retrieval time	Few seconds	10 s or more	1 min or more
Life expectancy	Excellent; May get corrupted	Excellent if handled properly	Finite
Relative cost	Very high	Low	Very low
Use	Online; Immediate access	Nearline; long-term archive; Disaster recovery	Offline; long-term archive; Disaster recovery

Table modified from reference 9

Traditional PACS archives have been based on a three-tier architecture, with fast, low-capacity storage for online storage; moderately fast, large-capacity storage for nearline storage; and a slower system with very large capacity for offline storage and backup. These systems had to rely on disproportionately large nearline storage, as magnetic discs had the highest cost per storage unit. Decreasing costs of magnetic disk storage have made the use of RAIDs increasingly popular. Most current storage systems employ a two-tier architecture: server-based storage to meet short-term needs for 6 months to 2 years and long-term archives for storage for longer periods and backup functions. A software application described as hierarchical storage management (HSM) automatically manages the migration of data between the online database and long-term storage.[6]

## Planning a data archival system

Before planning a data archival system an in-depth analysis of the workflow of different imaging modalities in the department and the average amount of data produced per study needs to be performed. With continuing advances in imaging technology, the number of radiological investigations as well as the amount of data generated per imaging study are constantly on the rise. Provisions must be made for newer, more advanced, imaging equipment that may be added to the department in the future. With a lot more ‘-logies’ such as cardiology, gastroenterology, ophthalmology, and pathology adding to the image data that needs archiving, the storage requirements of these departments also needs to be considered during planning.

The duration for which medical records and imaging data must be stored is dictated by hospital policies and local legal requirements. In general, 90% of all accessed data is less than a year old. Thus, it is wise to invest in at least a year's worth of online storage along with the PACS. Considering that the prices of magnetic disc-based storage will continue to fall, it may be prudent to keep adding additional storage space as the need arises. Longevity of the archiving technology should also be kept in mind. All storage devices are prone to failure over the long term. Current technology may become obsolete 5 years from now and it may be impossible to recover the archived data if the vendor no longer supports the equipment. Migration of data from one storage medium to another, with consequent PACS downtime, may be required when the next-generation archival media are implemented.

## Future

As PACS technology continues to evolve, newer architectures and storage media are being experimented with, in the attempt to provide faster and more efficient and cost-effective archival systems. Most current storage media are connected directly to the PACS server (direct attached storage). This architecture imposes restrictions on the number of drives that can be attached to the server and thus limits the scalability of the archive. Newer architectures such as network attached storage (NAS) and storage area network (SAN) offer advantages in the form of upgradeability, connectivity, and security. Similarly, newer technologies such as Blu-ray disc technology and holographic data storage (which records data in three dimensions, instead of just on the surface as in the optical disc) are finding their way into the PACS environment.[7] The latter offers yet another solution to the ever-increasing demand for data storage capacity and may prove to be an alternative to optical and magnetic storage systems in the future.
